# Parenthood and science careers: the impact is not the same for everyone

**DOI:** 10.1590/S2237-96222022000200013

**Published:** 2022-07-06

**Authors:** Pâmela Billig Mello Carpes, Fernanda Staniscuaski, Leticia de Oliveira, Rossana C. Soletti

**Affiliations:** 1 Universidade Federal do Pampa, Campus Uruguaiana, Uruguaiana, RS, Brazil; 2 Universidade Federal do Rio Grande do Sul, Departamento de Biologia Molecular e Biotecnologia, Porto Alegre, RS, Brazil; 3 Universidade Federal Fluminense, Departamento de Fisiologia e Farmacologia, Rio de Janeiro, RJ, Brazil; 4 Universidade Federal do Rio Grande do Sul, Departamento Interdisciplinar, Tramandaí, RS, Brazil

Gender inequality in science and the factors responsible for this phenomenon are being increasingly discussed and investigated. Worldwide, women’s participation in science is lower than that of men, decreasing even more in positions of power, leadership and decision-making.^1^ Although in Brazil we are moving towards a similar number of male and female scientists, progression in scientific careers is slower and more difficult for women. A variety of positions related to academia and science have never been held by women,^2^ so there are more women at the bottom of Brazilian scientific careers, while at the top, there are more men - the so-called scissor effect.^2^ ^,^ ^3^

The factors that help to understand this phenomenon are diverse, ranging from cultural determinants, related to the social role attributed to men and women, to issues related to explicit prejudice, harassment, various forms of violence and implicit biases, pushing women away from the hostile environment often found in academia.^4^ This set of multiple factors also involves motherhood.^5^ ^,^ ^6^ Parenthood brings great and different responsibilities, which can impact scientists’ careers, and the academic community should be aware of this impact, which is not the same for men and women.

## The Parent in Science Movement

The Parent in Science (PiS) Movement was created in 2016, with the goal of promoting the discussion about the impact of parenthood on the career of scientists in Brazil. Among the first actions of PiS was the creation of the #maternidadenolattes campaign, seeking the inclusion of a field on the Lattes curriculum to indicate maternity leave periods. The field was included in 2021.^7^

As a result of the mobilization and discussions motivated by the #maternidadenolattes campaign, the need to consider maternity leave periods when assessing the curricula of mother scientists has become more evident. With effect from 2019, several universities began to take maternity leave into account in their internal funding calls and postgraduate program applications. In general, the policy adopted has been to assess a longer period in the curriculum when analyzing productivity, or to use correction factors or additional scores when evaluating the curricula of scientists who are mothers. 

## Motherhood and science: what do the data say?

The PiS undertook its first survey between 2017 and 2018^5^ to investigate the impact of motherhood on the career of Brazilian women scientists. It revealed what many had already identified in their daily academic life: a great impact of motherhood on productivity. The results showed immediate repercussions on the productivity of mothers scientists, with a decrease in the number of scientific publications.^5^ This decrease is observed similarly in different fields, including the health sciences, and is not restricted to the maternity leave period - it appears to last for at least four years after the birth of the first child.^5^ The same scenario has also been found in other countries.^8^

These results suggest that motherhood has an important impact on female scientists’ careers, which is not a specific feature of academia but rather of work environments in general. Previous studies have indicated that motherhood leads to women being penalized, while fatherhood does not have the same consequence for the professional career of men. A study conducted in the United States and published in 2007 simulated job applications and compared the assessment of equally qualified male and female applicants, matching ‘gender’ and ‘race/skin color’, and found that the groups compared differed only in their parental status.^9^ The experiment revealed that mothers were penalized in the process, receiving, for example, a lower starting salary recommendation than women who did not have children.^9^ Fathers, on the other hand, were not penalized, and some even benefited from the status of being fathers.^9^

This last result is probably related to the stereotype that caring for children is mostly a woman’s responsibility - a social construct that ends up having repercussions on women’s professional careers. In Brazil, women are those most responsible for carrying out domestic chores and caring for people, dedicating twice as much time per week as men do to these activities.^10^ This demand on their time reduces women’s availability for other tasks, generates fatigue and stress and, therefore, harms their physical and mental health. Moreover, it is known that the academic-scientific working day often exceeds regular working hours, requiring additional time for writing and reviewing articles, reading and studying, mentoring students, etc. - additional time that is often not available in the routine of women who do both academic-scientific work and also take care of their homes and children. 

Although this configuration deserves to be questioned, women, especially mothers, are left behind. A commonly used fallacious argument is that “women are better at multitasking”. A study conducted in Germany in 2019, in which researchers tested the performance of men and women in different multitasking activities, found that there was no gender difference when it came to performance.^11^ In other words, the idea that women are capable of doing multiple things at the same time reflects a stereotype that helps to maintain gender biases in our society.

## Diverse Motherhoods

IIf motherhood alone impacts the career of mother scientists, the implication is even greater when other factors are considered. The interaction of factors that interfere with life in society, such as race/skin color, sexuality and disability, among other factors, is called intersectionality. A study released in 2022 analyzed millions of published scientific articles in order to study the relationship between scientists and the science they produce, as well as the relationship between scientists’ intersectional identities, their research topics and scientific impact.^12^ The authors showed that scientists from minority groups tended to publish in scientific areas and in research topics that reflected their social gender and race identities, while the participation of White authors in different research topics was balanced.^12^

With regard to race/skin color, it is well known that worldwide science is mostly done by White people.^13^ The participation of Black people is limited, especially when they are women.^13^ In Brazil, Black women account for only 3% of doctoral advisors.^14^ Gonzales and Harris discuss the assumption of incompetence of Black women in academia, which affects hiring, promotion and professional stability of these women, and influences on relationships with students, colleagues and administrators.^15^ These women are strongly impacted by stereotypes related to race, so it is essential to establish support networks to transform the work environment.^15^ In addition, racial and gender issues are associated with motherhood, so Black mothers face multiple biases,^16^ and an even greater impact on their academic career.^17^

Regarding disabilities, there are two main situations to be considered: scientists with disabilities, and those who are parents of people with disabilities. there are scientists with disabilities in many areas of knowledge; however, these people are underrepresented compared to their numbers in the general population^18^ and this is not related to lack of interest or skills.^19^ Disability - and the discrimination that comes with it - in a context of high demands and competitiveness as found in academia, helps to explain this underrepresentation.^20^ Although some institutions adopt support policies aimed at people with disabilities, ableism, stigmatization and disabling barriers hinder their participation.^20^ Another aspect to consider, in intersectionality with parenthood, is the situation of parents of children with disabilities. Mothers are the main, and in many cases the only, caregivers of children with disabilities.^21^ ^,^ ^22^

In these situations, in which children often require constant care for many years, the impact on careers, especially for mother scientists, can be even greater. Currently, there is a bill of law in progress in the Senate (Bill No. 242/2020), which establishes the extension of maternity leave for a further 180 days, as well as provisional job stability in the case of caregivers of newborns with disabilities.^23^ This policy is important so that the family can organize and adjust to the new routine. Maternity support policies in science should consider that mothers of children with disabilities experience an even more significant impact on their scientific career and productivity. Support strategies should be considered, such as permanent compensation with regard to assessment of time when evaluating the curricula of these mothers, compared to others, since children with disabilities may require prolonged care, sometimes lasting for their entire lifetime.

Sexuality is another aspect worthy of attention. A survey conducted in the United Kingdom revealed that 18% of scientists who are gay, lesbian, bisexual or transgender, as well as other sexual and gender minorities (LGBTQIA+), reported experiencing harassment, bullying or some type of exclusionary behavior in the workplace; this number rises to 32% in the case of transgender people and those who do not identify as male or female (non-binary).^24^ When parenthood is included in the discussion, for this group especially, the topic needs to be discussed beyond professional or scientific activities; issues related to LGBTQIA+ people must be discussed more in society, to promote parental empowerment and the welcoming of these fathers and mothers.^25^ Statistics on LGBTQIA+ people who have children are still unknown in most countries, as the data collected rarely includes the gender or sexual orientation of pregnant women or their partners.^26^ The same applies in the case of science; information about the sexuality of scientists is scarce. These data are essential to the proposition of effective diversity policies in science.

## Challenges

To consider the impact of motherhood on science, and in particular, intersectionality is in itself a huge challenge. Beyond this, the COVID-19 pandemic has impacted the work of scientists around the world, and yet again, this impact has not been equal for everyone. The decrease in the number of new projects that began to be conducted in 2020, in the context of the pandemic, has been most pronounced among female scientists and those with young children from 0 to 5 years of age.^27^ In Brazil, a PiS survey ratified these data, showing that Brazilian mother scientists and Black female scientists, regardless of motherhood, have had greater difficulty in continuing to submit papers during the pandemic period.^17^ These data are indicative of the even greater challenges that women will face in the post-pandemic period.

**Figure 1:**
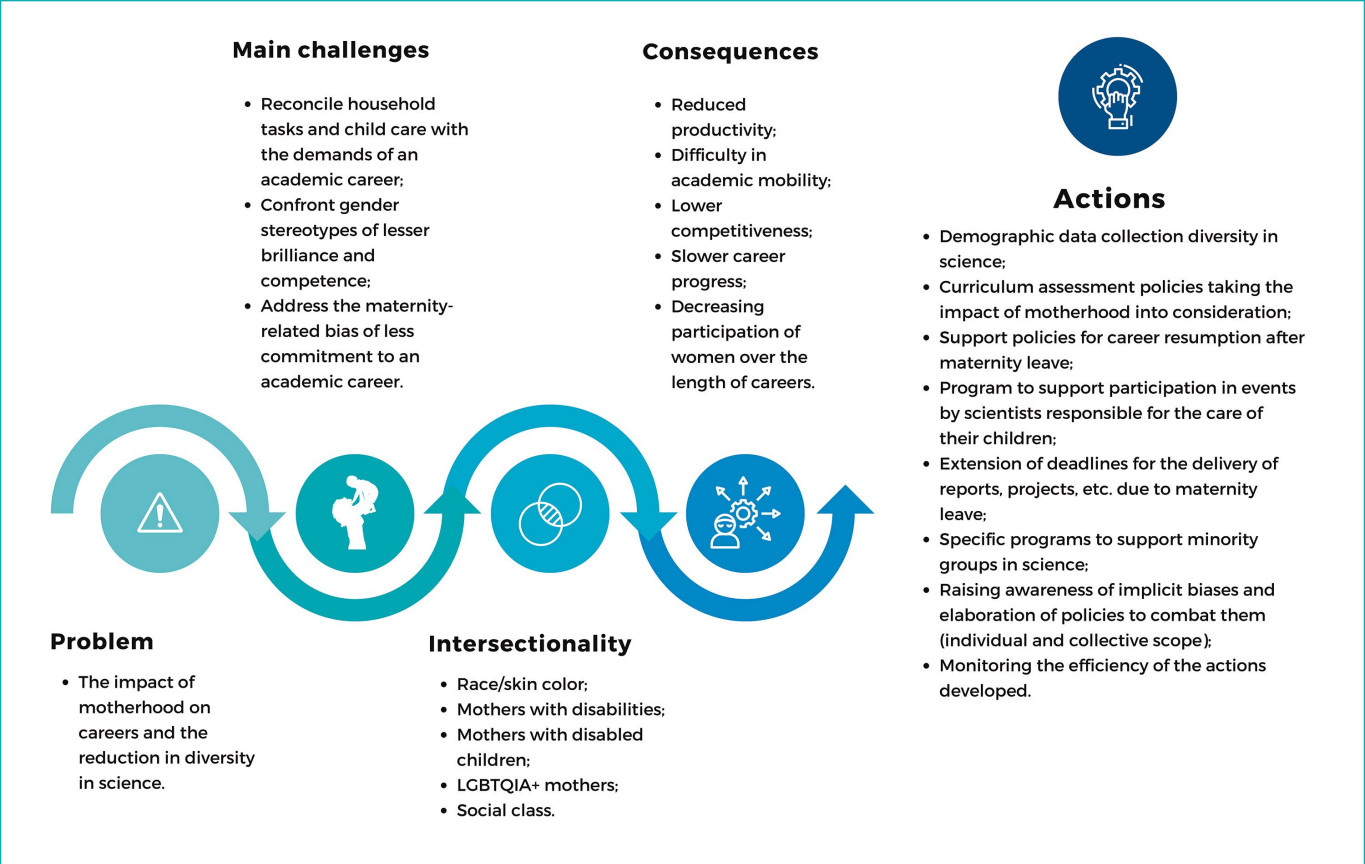
Examples of actions to mitigate the impacts of motherhood on the careers of women scientists

Note: Motherhood impacts women scientists’ careers, bringing many challenges. This impact is even greater when intersectional factors are considered. As a consequence, there is a series of events that culminate in fewer women in academic careers. Actions are needed to ensure the participation of mother scientists and greater diversity in science.

Gradually, face-to-face activities are being resumed in Brazilian education and research institutions, and effective actions to mitigate the negative impacts of the pandemic are fundamental, especially in the careers of women who are mothers.^28^ Recently, the PiS has highlighted the importance of this and suggested some actions,^29^ which, along with others, are exemplified in Figure 1.

It is essential to value diversity in science, going beyond speech! Diversity positively impacts the capacity for innovation and increases the creative capacity of a research team.^30^ Ensuring more diverse teams is not only a matter of fighting for the rights of people to be where they want to be, but it is also a fight for better science.
